# Broken Doses and the Repetition of the Dose

**Published:** 1892-10

**Authors:** 


### HOMŒOPATHIC PHYSICIAN,

A MONTHLY JOURNAL OF

HOMŒOPATHIC MATERIA MEDICA AND CLINICAL MEDICINE.

"If our school ever gives up the strict inductive method of Hahnemann, we are lost, and deserve only to be mentioned as a caricature in the history of medicine."—CONSTANTINE HERING.

Vol. XII. OCTOBER, 1892. No. 10.

### EDITORIAL.

BROKEN DOSES AND THE REPETITION OF THE DOSE.—At the meeting of the Internationals there was a prolonged and interesting discussion upon broken doses suggested by the paper of Dr. Arthur G. Allen. This paper and the discussion appear in full in this number of THE HOMŒOPATHIC PHYSICIAN. This discussion revives the old question of the repetition of the dose, and a careful reading of what is there said will give an intelligent idea of the variety of opinions held upon this subject of repeating.

After all, the selection of the ideal simillimum for any given case is the only true solution of the problem.

When a remedy is given that is the exact simillimum of the case to be treated there is no appreciable time between the administration of the first dose upon the tongue and the beginning of the cure. Homœopathic physicians have often been heard to say that the medicine no sooner touched the patient's tongue than the symptoms began to ameliorate. The late Dr. Lippe, who was remarkable for his capacity to select the simillimum, has repeatedly expressed himself thus in the hearing of the editor. Other physicians have had a similar experience, and many who read these lines will have recalled to their memory with pleasure observations of like character. Now, then, if our remedies were fully proven, our repertories perfect, and our skill in examining the case higher, we would more frequently realize the deep satisfaction of finding a medicine that has the totality of the symptoms of the sick condition which it is our aim to relieve, and more frequently witness these miraculously prompt cures. The further we diverge from such mathematical requirements as constitute the homœopathic formula of cure, the more we must repeat, the more we must change the prescription, the more will our views vary as to what are the requirements in general, and the more conflicting will be the testimony of our individual experiences.

The deeper we look into the operation of these three principles—the simillimum, the minimum, the single remedy—the more difficult will appear its requirements and the richer the rewards to such as can patiently and industriously penetrate these depths and overcome these difficulties. No more helpful way of getting an insight into these depths of possibility for suffering humanity can be devised than the reading of thoughtful papers by sincere Hahnemannians before assemblages of their brethren and thus provoking discussion.

